# Characterizing and Optimizing Piezoelectric Response of ZnO Nanowire/PMMA Composite-Based Sensor

**DOI:** 10.3390/nano11071712

**Published:** 2021-06-29

**Authors:** Xiaoting Zhang, Jose Villafuerte, Vincent Consonni, Jean-Fabien Capsal, Pierre-Jean Cottinet, Lionel Petit, Minh-Quyen Le

**Affiliations:** 1 Electrical Department, Ladoua Campus, University Lyon, INSA-Lyon, LGEF, EA682, F-69621 Villeurbanne, France; xiaoting.zhang@insa-lyon.fr (X.Z.); jean-fabien.capsal@insa-lyon.fr (J.-F.C.); pierre-jean.cottinet@insa-lyon.fr (P.-J.C.); lionel.petit@insa-lyon.fr (L.P.); 2 University Grenoble Alpes (iCampus), CNRS, Grenoble INP, LMGP, NanoMAT Team, F-38000 Grenoble, France; jose.villafuerte@grenoble-inp.fr (J.V.); vincent.consonni@grenoble-inp.fr (V.C.); 3 University Grenoble Alpes (iCampus), CNRS, Grenoble INP, Institute NEEL, F-38000 Grenoble, France

**Keywords:** ZnO nanowires composite, piezoelectric sensor, electrical and piezoelectric characterizations, finite element method, medical application

## Abstract

Due to the outstanding coupling between piezoelectric and semiconducting properties of zinc oxide nanowires, ZnO NW-based structures have been demonstrating promising potential with respect to their applicability in piezoelectric, piezotronic and piezo-phototronic devices. Particularly considering their biocompatibility and biosafety for applications regarding implantable medical detection, this paper proposed a new concept of piezoelectric composite, i.e., one consisting of vertically aligned ZnO NW arrays and an insulating polymer matrix. First, the finite element method (FEM) was employed to drive optimization strategies through adjustment of the key parameters such as Young’s modules and the dielectric constant of the dielectric constituents, together with the density and dimension of nanowire (NW) itself. Second, to investigate the functionality of each individual layer of composite, different designed structures were fabricated and characterized in terms of electrical and piezoelectric properties. Next, experimental and simulation tests were performed, indicating that the decreasing thickness of the top poly(methyl methacrylate) layer (PMMA) can substantially enhance the piezoelectric sensitivity of the ZnO NW composite. Besides the further benefit of no polarization being needed, our material has a comparable charge coefficient (d_33_) with respect to other lead-free alternatives (e.g., BaTiO_3_), confirming the high sensing abilities of the developed structure based on vertically aligned ZnO NW arrays. Finally, a time-varying model combining piezoelectricity and electric circuit modules was investigated in detail, giving rise to an estimation of the d_33_ coefficient for ZnO NWs. Based on this study, the developed material is revealed to be highly promising in medical applications, particularly regarding the FFR technique, where coronary pressure can be measured through a piezoelectric sensor.

## 1. Introduction

### 1.1. Piezoelectric Materials Based on ZnO Nanowires

With developed nanocrystal synthesis technology, nanowire-based systems have been researched for a wide range of applications in optoelectronics, actuation, electronics, sensors, and electromechanics [1,2]. In comparison to bulk materials, nanowires (NWs) give rise to remarkable, novel, electrical, mechanical, chemical and optical properties, owing to their high surface-to-volume ratio, relevant to surface and quantum confinement, for instance. Thus, NWs are integrated into multi-functional systems with versatile properties, such as the single NW lasers [3], photovoltaic devices [4], nanosensors [5], single NW spectrometers [6], optical applications [7,8], and so on. Among these applications, recently, ZnO NWs possessing a non-centrosymmetric wurtzite structure have taken advantage of the strong direct piezoelectric effect, resulting in great attention among the scientific communities for their considerable superiorities. Firstly, by utilizing the coupling of semiconducting and piezoelectric properties, ZnO is one of the most promising materials, with attractive properties like direct wide-bandgap energy, large excitation binding energy, and high electron mobility [9]. Moreover, concerning the piezoelectric behavior, ZnO does not require a high-voltage poling process, as compared to piezo-ceramic lead zirconate titanate (PZT) [10], barium titanate (BaTiO_3_) [11], and poly (vinylidene fluoride) (PVDF) films [12,13,14,15]. More importantly, ZnO is also a biocompatible, non-toxic material, which further broadens its applications in the healthcare arena, especially for in vivo biosensing and bio-detection tools [16,17].

In addition, concerning the limitation of the size requirement in portable electric devices, ZnO NW-based piezoelectric devices can be regarded as self-powered systems that employ energy-harvesting from their environment to drive electronic devices without an external power source [18]. Thus, they provide the possibility of miniaturization capabilities, especially in an implantable biomedical device. Indeed, according to the different purposes, ZnO NW/polymer composites can be categorized into nanogenerators (NGs), harvesting random mechanical energy converted into electric energy, and sensors detecting the dynamic mechanical deformation. Wang et al. have firstly demonstrated the excellent energy-harvesting properties in vertically integrated nanogenerators (VING), where one end is attached to the substrate and the other is free to move [19,20]. Several studies have also reported on the development of flexible laterally integrated NGs, based on a cyclic stretching–releasing movement where both ends are fixed [21,22]. Up until now, many publications have concentrated on exploiting the piezoelectric output of ZnO VING [2]. However, few of them explored the feasibility of flexible sensors based on vertically aligned ZnO NW arrays and providing a reliable method of characterization for these materials [23,24,25].

Researchers have demonstrated the piezoelectric coefficient (d_33_) of 12 pC/N for lithium-doped ZnO single crystals, and, by theoretical computation, a d_33_ = 8 pC/N for undoped ZnO with a wurtzite structure [26,27]. It was believed that the ZnO NWs should have larger piezoelectric constants as opposed to bulk ZnO, due to the nanoscale surface effects. However, at such a nanoscale, it is somehow difficult to accurately measure the piezoelectric coefficient, due to small, generated charges and voltages. Wang et al. firstly demonstrated the experimental measurement of piezoelectric generation from bent ZnO NWs via the atomic force microscopy (AFM) technique [28,29,30]. Zhao et al. found that, by utilizing piezoresponse force microscopy (PFM), the effective piezoelectric coefficient (d_33_) of a ZnO nanobelt varies from 14.3 pC/N to 26.7 pC/N as the driving frequency is decreased [31]. However, for single ZnO nanopillars with 300 nm diameter and 2 µm length, the d_33_ value was measured to be around 7.5 pC/N, slightly lower than that found in the bulk, which might be due to structural defects [32]. To give a rough estimation of the effective piezoelectric coefficient d_33_ of ZnO NW arrays, we propose in this work an alternative approach based on COMSOL simulation-based FEM, which is then fitted with realistic experimental data.

### 1.2. Motivation of This Work

**Figure 1:**
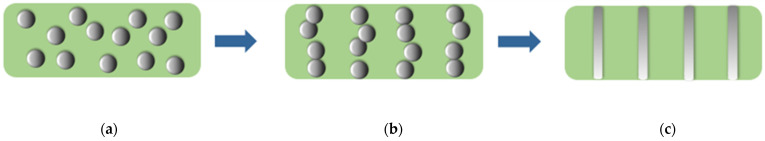
Different architectures of the ZnO/PMMA composite: (**a**) particles randomly dispersed; (**b**) aligned particles; and (**c**) vertically aligned NW arrays within the polymer matrix.

Our previous works pointed out that ZnO composites with a particle-aligned structure lead to substantial enhancement in the piezoelectric properties, as opposed to those composites with particles randomly dispersed [33,34] (see Figure 1a). However, the d_33_ coefficient of such materials, even in the best configuration (i.e., high concentration and perfect alignment, as illustrated in Figure 1b), was still extremely low (d_33_ = 0.5 pC/N) compared to composites filled with classical piezoelectric particles like either PZT (d_33_~20 pC/N) [35] or BaTiO_3_ (d_33_~5 pC/N) [36]. This work aims to demonstrate that, based on the vertically aligned ZnO NW arrays whose filler density and alignment structure are considered to be optimized (cf. Figure 1c), it is possible to substantially boost the piezoelectric sensitivity. Our experimental results reveal that the d_33_ coefficient of ZnO NW composite can reach approximately 3.5 pC/N, which is comparable to its conventional BaTiO_3_ counterpart. Optimization in terms of design and process could further enhance the properties of a ZnO composite, confirming its feasibility in applications such as sensing devices, especially in a healthcare context.

The first objective of this research focuses on the implementation of numerical simulations based on COMSOL Multiphysics, combining the electrical and piezoelectric characteristics of ZnO NW/polymer composite. The tested model designed with vertically aligned ZnO NW arrays are operated in a mechanical compression mode. Optimization guidelines are found to enhance the piezoelectric sensitivity relying on the best tuning of material properties as well as structural design. Our results suggest that the performance of a ZnO NW composite should be considerably affected by various parameters, including the mechanical and dielectric features of the polymer matrix, together with the density and dimension of NW arrays. Another objective of this work focuses on characterizing the electrical and piezoelectric responses of the developed sensor via an experimental test bench. To further explore the function of each layer, samples with various compositions were fabricated and compared together. The properties of ZnO nanowires are considered on the basis of a thorough and comprehensive analysis of realistic empirical data. Finally, a validation on the different thickness of the top layer performed in both experiment and simulation allows us to predict the theoretical piezoelectric coefficient (d_33_). In particular, the optimal thickness that would lead to good piezoelectric sensitivity is deduced from the best compromise of material characteristics.

Our target application for the developed sensing device uses the fractional flow reserve (FFR) technique to evaluate the significance of artery narrowing, particularly in coronary stenoses. FFR is defined as the maximum myocardial blood flow in the presence of stenosis divided by the theoretical maximum flow in the absence of stenosis [37]. The existing pressure sensor technologies employed in FFR measurements are optical, capacitive, and piezoresistive [38]. All of them show some limitations. An optical sensor is advantageous, due to its small size and ability to withstand environmental effects, and is extremely sensitive to the bending of the fibers. Indeed, imposing stiffness to the catheter provokes limitations in the case of tortuous vessels [38,39]. Capacitive sensors, characterized by easy and cheap processability, high sensibility, and lower power consumption, require complex readout circuitry [40]. Finally, piezoresistive sensors offer mechanical stability, but at the same time, they show critical drawbacks, like a high power requirement, large temperature dependence offset, non-linearity, long-term instability in dynamic field conditions, and impart undesirable stiffness to catheters [41]. We believe that ZnO NW-based piezoelectric composites would be an alternative solution as an implantable and biocompatible pressure sensor, and thus be more easily implemented on a catheter for FFR pressure measurement.

Therefore, this study aims to bring a full characterization of the electrical, dielectric and piezoelectric properties of ZnO NW/polymer composite. Experimental tests, together with simulation models, were developed to identify relevant parameters, which can substantially affect the piezoelectric sensitivity of the developed material. This, to some extent, is not easy because of the complex material structure that consists of several layers. From the point of view of practical applications, the characterization of each layer involved in this study is critically important, leading to a better understanding of the behavior of the whole sample’s structure. Finally, the proposed material is revealed to be promising for medical use, particularly for pressure measurement in the FFR technique.

## 2. Fabrication and Method of Characterization

### 2.1. Synthesis of ZnO Seed Layer and ZnO Nanowires

**Figure 2:**
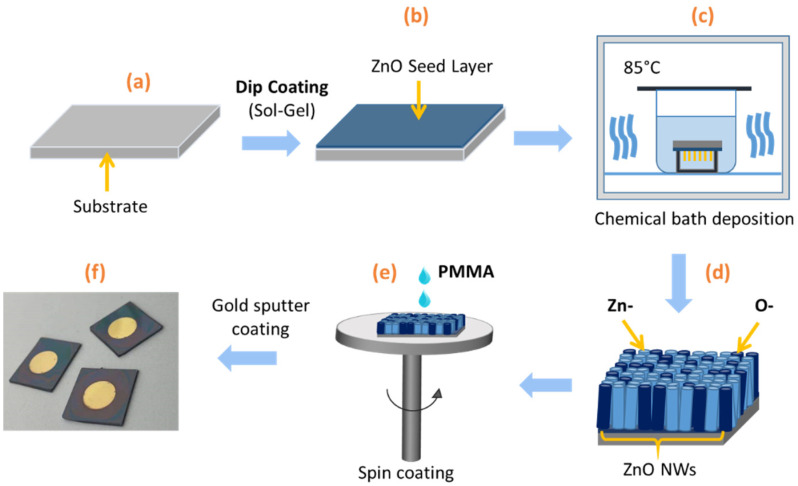
Fabrication steps of ZnO NW arrays/PMMA composite : (**a**) Preparation of Si substrate; (**b**) Deposition of ZnO seed layer (SL); (**c**) Growth of ZnO NWs through chemical bath deposition (CBD); (**d**) Representation of different polarities of ZnO NWs; (**e**) Incorporation of PMMA dielectric polymer; (**f**) Final sample coated with circular gold electrodes.

In this work, we designed a typical structure consisting of vertically aligned ZnO NW arrays, encapsulated in a stiff polymer matrix. A ZnO thin seed layer (SL) was deposited on a silicon (Si) substrate, above which the vertically aligned ZnO NWs grew. All fabrication steps were described in Figure 2. Firstly, the samples were prepared on a 675 ± 25 µm-thick (001) Si substrate, cleaned with acetone and isopropyl alcohol in an ultrasonic bath (Figure 2a). Secondly, a 40 nm-thick ZnO SL, using a sol-gel process, was prepared with 375 mM zinc acetate dehydrate (Zn(CH_3_COO)_2_·2H_2_O, Sigma-Aldrich, Darmstadt, Germany) and 375 mM monoethanolamine (MEA) (Sigma-Aldrich, Darmstadt, Germany) mixed in pure ethanol (Figure 2b). Afterward, continuous stirring was performed for several hours at 60 °C and then at room temperature. The Si substrates were dipped into the solution under a controlled atmosphere (<15% hygrometry). After this, they were annealed at 300 °C for 10 min on a hot plate for the evaporation of residual organic compounds, and at 500 °C for 1 h in an oven under air for the crystallization of SL layers. Thirdly, ZnO NWs were grown by chemical bath deposition (CBD) in a sealed reactor containing an equimolar proportion of the precursors, 30 mM zinc nitrate hexahydrate (Zn(NO_3_)_2_·6H_2_O, Sigma-Aldrich, Darmstadt, Germany), and hexamethylenetetramine (HMTA, Sigma-Aldrich, Darmstadt, Germany) mixed in deionized water (Figure 2c). The sealed reactor was kept for 3 h in an oven at 85 °C. From each nucleation site created by a polycrystalline film deposition of both Zn- and O-polar seeds, there is a ZnO NW with the same polarity of the site from whence it grew. The duality of colors represents the two different polarities. For instance, as described in Figure 2d, the light blue corresponds to the Zn-polarity, while the dark blue denotes the O-polarity. It is noteworthy that this figure is used for illustration purposes only. A real scale and exact amount (or density) of NWs can be deduced from scanning electron microscopy (FESEM) imaging (cf. Section 3.1). Next, different film thicknesses of poly(methyl methacrylate) (PMMA with 8% solids, All Resist) were spin-coated on top of the different devices (Figure 2e). To improve the robustness and stabilize the NW structure, a PMMA matrix was spin-coated as interstitial insulating material among NWs. With the aim of preventing electrical leakages and direct contact between NWs and the top electrode, a dielectric top layer (e.g., made of PMMA polymer) was stacked on the ZnO NW arrays. Lastly, to create electric contact, a circular gold coating with an 8-mm diameter and approximately 30-nm thick was deposited on both sides with a sputter coater (Cressington, 208HR, Watford WD19 4BX, UK) (see Figure 2f).

### 2.2. Characterization Methods

#### 2.2.1. Dielectric Characterization

Broadband dielectric spectroscopy was measured utilizing a frequency response analyzer (Solartron, 1255, Oak Ridge, TN, USA) together with a dielectric interface (Solartron, 1296A, Oak Ridge, TN, USA) at ambient temperature. Each composite was clamped in a sample holder (AMETEK, 12962a, Massy, France). An alternating voltage with an RMS value of 200 mV was applied to derive the precise measurement of dielectric spectra, ranging from 1 Hz to 1 MHz.

#### 2.2.2. Piezoelectric and Electrical Characterizations under Dynamic Mechanical Excitation

**Figure 3:**
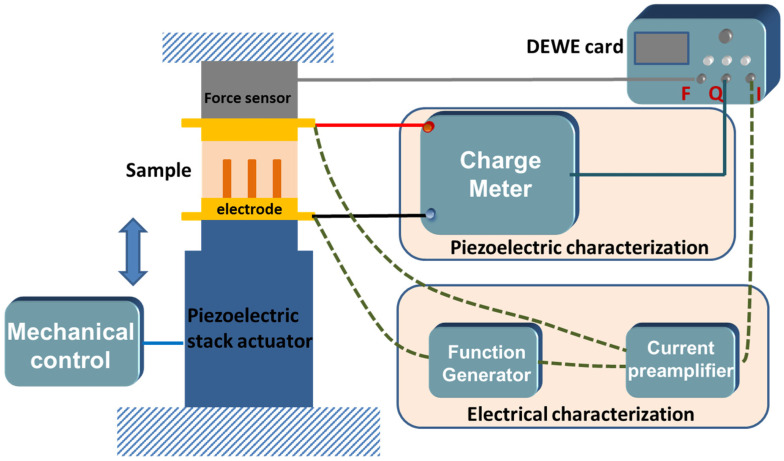
Schematics of an experimental setup for piezoelectric and electrical characterizations.

The piezoelectric and electrical characterizations of samples under external vertical dynamic mechanical excitation were performed using a sensitivity setup, as shown in Figure 3.

Regarding the piezoelectric test, the main setup includes a piezoelectric stack actuator (PI 246-50, Karlsruhe, Germany) force sensor (HBM, Darmstadt Germany), and two printed electrode circuit boards. The sample was clamped between two electrodes and aligned with the other parts to keep high perpendicularity. The piezoelectric stack actuator was controlled by a waveform generator (Agilent, 33500B, Santa Clara, CA, USA) together with a voltage amplifier (Trek, Model 20/20C), offering a dynamical mechanical excitation to samples. Meanwhile, the compressive force exerting on the sample was recorded via a DEWE card (Sirius, 8XSGT, SI-1420 Trbovlje, Slovenia) which was connected to a computer. During the experiment, the mechanical frequency of 1 Hz was fixed, and various amplitudes of force were applied to explore the influence of external stress. During the mechanical movement, the piezoelectric charge (*Q*) was collected in a high-sensitivity charge meter (KISTLER, Type 5015, Winterthur, Switzerland) through a short-circuit loop. Therefore, the charge coefficient d_33_ can be defined as the ratio of the charge density to the applied stress:

(1) $$d_{33}=\frac{D}{T}=\frac{\frac{\Delta Q}{S_{ative}}}{\frac{\Delta F}{S}}$$

where *T* and *F* represent the mechanical stress and applied force, respectively; *S_active_* and *S* denote the surface of the gold electrode and the sample, respectively; *D* is the electric displacement (or charge density).

To establish the electrical properties of a sample subjected to a mechanical solicitation, both current and voltage signals were simultaneously recorded. Firstly, an AC input voltage was applied to the electrodes of the sample by means of a waveform generator (Agilent, 33210A). Secondly, the resulting current was detected by a low-noise current preamplifier (Stanford Research Systems, SR570) with a gain setting from 100–500 µA/V. Finally, the characteristic loop of current density (*J*) versus electric field (*E*) was monitored with the corresponding force through the DEWE interface. Post-data treatment was performed via the Origin software.

## 3. COMSOL Simulation

In this section, a COMSOL Multiphysics-based FEM was used to simulate a fully coupled electromechanical system. The FEM simulations can provide a clear understanding of the working principle, and in turn, facilitate the optimization of output efficiency through the relevant parameters.

### 3.1. Unit Cell Model of ZnO Nanowire

#### 3.1.1. Structural Design

**Figure 4:**
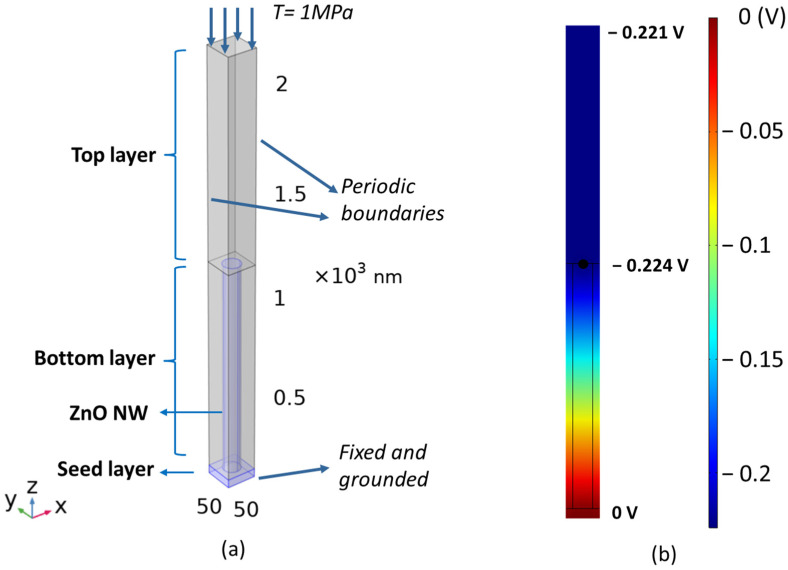
COMSOL model of ZnO NW: (**a**) schematic structure of a unit cell consisting of ZnO SL, bottom matrix layer, top matrix layer, and single ZnO NW with proper boundary conditions; (**b**) electric potential longitudinal profile of unit cell subjected to an axial compression (viewed in 2D YZ cut plane).

In order to simplify the numerical model and reduce the computational time, a typical unit cell with periodical boundary conditions on the sidewalls of ZnO NWs was defined, as depicted in Figure 4a. In the bottom layer, a 3D-cylinder shape representing a ZnO NW with 42 nm diameter and 1031 nm length was chosen, on a 40 nm-thick SL, and embedded in a PMMA matrix. A dielectric top layer, with a varying thickness initially set to 1000 nm, was stacked above the bottom layer to create a unit cell with a size of 57 × 57 × 2071 nm^3^ that matches the realistic NW density (51 ± 7 µm^−2^). The material properties of ZnO and PMMA were taken from the COMSOL material library. Regarding the boundary conditions, a fixed mechanical constraint and electrical ground condition were applied at the bottom of the SL, while the top of PMMA was subjected to a boundary stress T=1 MPa along the *z*-axis.

The following coupled equations for piezoelectric-based material can be solved using a COMSOL Multiphysics FEM:

(2) $$S_{3}=s_{33}T_{3}+d_{33}E_{3}$$

(3) $$D_{3}=d_{33}T_{3}+\varepsilon_{33}E_{3}$$

where *T*_3_ is the mechanical compression stress, *S*_3_ is the strain, *E*_3_ and *D*_3_ are the electric field strength and the electric displacement, respectively. $s_{33}$ $\varepsilon_{33}$, and $d_{33}$ denote the compliance, the dielectric constant, and the piezoelectric coefficient for strain-charge conversion, respectively. Indices 3 refer to the direction along the thickness’s sample.

More specifically, the external mechanical input is transformed into an internal stress applied to the piezoelectric composite. Then, the electric bound charges are generated on both ends of NWs and create a dipole-like potential along the *z*-axis. Thus, electric charges are transported to the top and bottom contacts, and, in turn, drive electron displacement. The evolution of the electrical potential generated in a unit cell is displayed in Figure 4b with a cross-section view. Under the application of compressive stress, the output piezoelectric potential generated along the NW becomes negative and its absolute value gradually increases from the bottom (connected to the ground) to the top. Interestingly, a very small variation (decreasing from 0.224 V to 0.221 V) of the potential occurs in the dielectric top layer, which probably results from the polarization of dielectric and electrostatic effects. With the aim of optimizing the material design and architecture, in the next steps, we focus on the influence of essential parameters, such as Young’s modulus and the relative permittivity of the dielectric polymer, together with the density and dimension of the ZnO NW.

#### 3.1.2. Influence of Young’s Modulus

**Figure 5:**
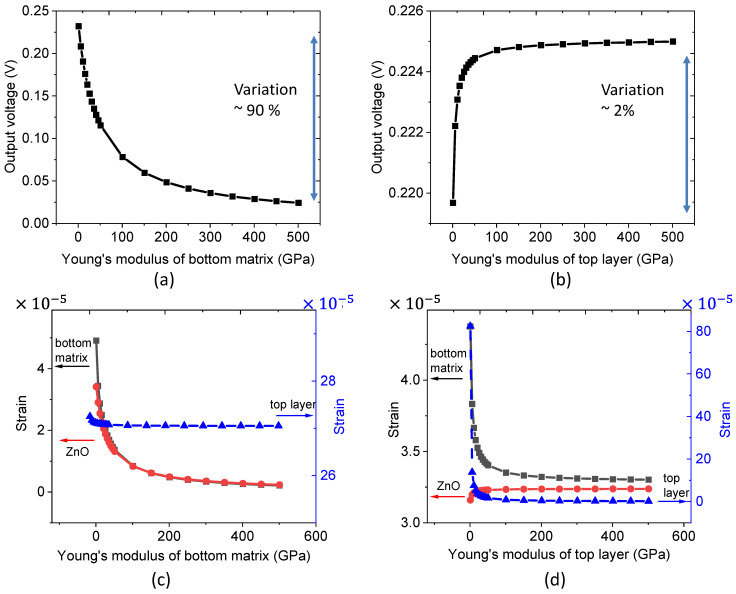
Effect of Young’s modulus of the bottom matrix and the top layer, respectively on: (**a**,**b**) the generated output voltage; (**c**,**d**) the resulting strain along the stress direction of different layers.

**Figure 6:**
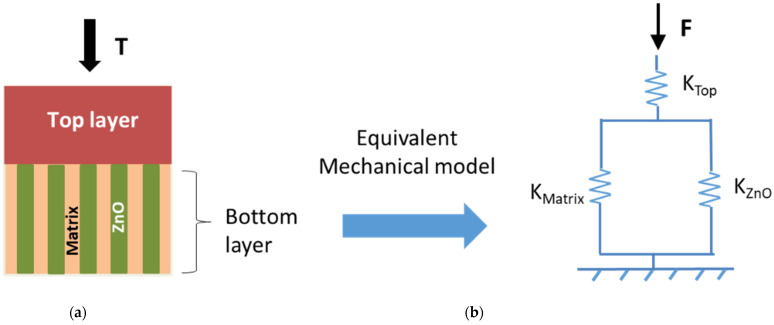
Equivalent mechanical schemes: (**a**) ideal fiber composite material; and (**b**) equivalent spring model.

As shown in Figure 5a, when increasing Young’s modulus of the bottom matrix (*Y_matrix_*) from 1 to 500 GPa, the output voltage drastically decreases under a 1 MPa compressive stress (*T*). This phenomenon could be explained on the basis of the ideal fiber composite material combining the iso-strain or iso-stress models [42]. Figure 6a describes the material structure where the arrangement of two elements (ZnO and the matrix) in the bottom composite layer is considered to be parallel with respect to the externally applied stress. Considering the stress distribution is homogenous to each layer:

(4) $$T=T_{top}=T_{bottom}$$

The strain response of the parallel structure along the force direction can be given by:

(5) $$S_{ZnO}=S_{matrix}=S_{bottom}=\frac{T}{Y_{bottom}}$$

According to Equation (5), the higher Young’s modulus of the bottom composite layer ($Y_{bottom}$), the lower the strain ($S_{bottom}$) that can be observed. This result is also confirmed in Figure 5c, where $S_{matrix}$ and $S_{ZnO}$ are identical and inversely proportional to $Y_{bottom}$. The behavior of the strain response with respect to Young’s modulus of the bottom layer allows us to explain the decreasing trend of the output potential shown in Figure 5a. As expected, no change in the strain of the top layer ($S_{top}$) is revealed, as its Young’s modulus remained constant.

In the following, we demonstrate that the mechanical structure of the composite can be featured as the spring model displayed in Figure 6b. Regarding the stress expression of the bottom layer:

(6) $$S_{bottom}=\frac{\Delta L_{bottom}}{L_{bottom}}=\frac{T}{Y_{bottom}}$$

where $L_{bottom}$ denotes the height of the bottom layer, and $\Delta L_{bottom}$ refers to its deformation, which is deduced by:

(7) $$\Delta L_{bottom}=\frac{T\times L}{Y}=\frac{F}{\frac{A*Y}{L}}$$

Given that $K_{bottom}=\frac{A*Y}{L}$ , where $K_{bottom}$ is the stiffness of the bottom layer and *A* is the surface, it follows that:

(8) $$\Delta L_{ZnO}=\frac{F}{K_{bottom}}=\frac{F}{K_{matrix}+K_{ZnO}}$$

Equation (8) allows us to confirm the equivalent mechanical model drawn in Figure 6b, which consists of a spring $K_{top}$ in parallel to a spring $K_{bottom}$ (i.e., corresponding to two parallel springs of ZnO and the matrix).

In Figure 5b,d, we fix Young’s modulus of the bottom matrix while varying the one of the top layer. Logically, increasing $Y_{top}$ leads to a decrease in the strain response of the top layer ($S_{top}$), i.e., tends to zero when $Y_{top}$ is high enough. Unexpectedly, in Figure 5d, there are some variations in the $S_{ZnO}$ and $S_{matrix}$, which would not theoretically be influenced by the variation of Young’s modulus of the top layer. Such variations are only obvious when a soft matrix top layer is made (low $Y_{top}$). This phenomenon is attributed to the non-homogeneous distribution of the strain near the NW and of the stress of each layer, particularly in the 3D model [43]. Contrary to the ideal 2D model illustrated in Figure 6, the stress is considered to be identical for both layers (Equation (4)), and the strain was the same for the polymer matrix and the ZnO NW (Equation (5)). In the case of a hard top layer (high $Y_{top}$), the composite can be assumed as an ideal model where the strain responses $S_{ZnO}$ and $S_{matrix}$ are close together and remain constant, despite the significant change in Young’s modulus of the top layer. These observations explain why the output voltage slightly increases with a soft top layer but is otherwise unchanged (Figure 5b). As the voltage variation is very small (~1%), it is reasonable to conclude that $Y_{top}$ has no noteworthy consequence on the piezoelectric output. From the above two simulations, considering the same variation range of Young’s modulus for the top and the bottom matrix layers, a decrease in the $Y_{matrix}$ gives a much higher efficiency for the optimization of the output voltage, as compared to an increase in $Y_{top}$.

#### 3.1.3. Influence of Dielectric Permittivity

**Figure 7:**
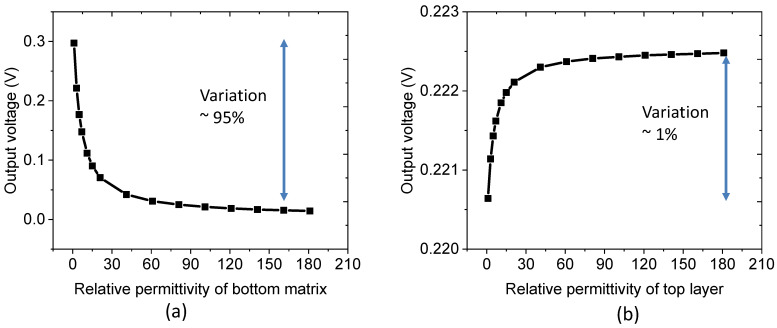
Effect of relative permittivity of the (**a**) bottom matrix layer, (**b**) top layer on the generated output voltage.

The effect of relative permittivity on the potential generation is illustrated in Figure 7. Indeed, the permittivity reflects the reduction of an effective electric field due to the presence of polarization in the dielectric medium. The higher the relative permittivity, the greater the resistance could be encountered during the formation of an internal electric field. As expected in Figure 7a, the output voltage significantly decreases with the increased relative permittivity of the bottom matrix layer ($\varepsilon_{matrix}$). Another explanation lies with the electric flux density ($D_{bottom}=\varepsilon E_{bottom}$) on the ZnO NW and matrix bottom layer. More specifically, because of the invariable normal electric flux density on the material, a higher $\varepsilon_{matrix}$ leads to a lower electric field and thereby decreases the electric potential transmission within the NW composite. Figure 7b shows the evolution of the output voltage as a function of the relative permittivity of the top layer ($\varepsilon_{top}$). A small increase of around 1% has been observed, confirming no significant effect of $\varepsilon_{top}$ on the piezoelectric response of the composite.

In conclusion, the best configuration, based on the consideration of Young’s modulus and dielectric permittivity, is a hard-top layer with high permittivity, and a soft bottom layer with low permittivity. It has been demonstrated that Si_3_N_4_ and Al_2_O_3_ are regarded as promising candidates for top insulating layers in a vertical integrated nano-generator structure [43,44]. Concerning the bottom layer, PMMA seems to be the favorable polymer thanks to its easy process, interesting dielectric and mechanical characteristics, as well as its high biocompatibility that makes it fascinating for medical applications. It is pointed out in this study that the properties of the top layer have less effect on the piezoelectric response of the composite. Thus, in the fabrication process, we employed PMMA directly as a top layer, for the sake of simplicity.

#### 3.1.4. Influence of NW Density

**Figure 8:**
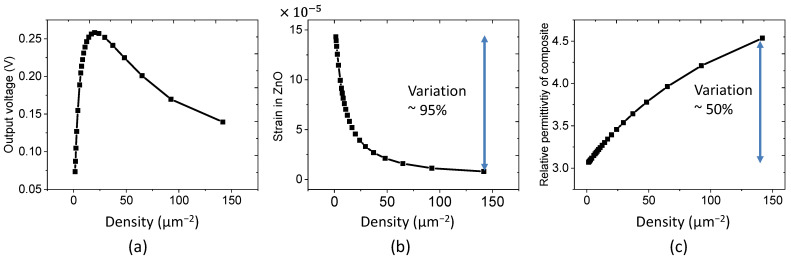
Effect of ZnO NW density on: (**a**) the generated output voltage; (**b**) the strain in ZnO along the stress direction; and (**c**) the relative permittivity of the composite.

The simulation test performed in Figure 8a allowed us to assess the relationship between the NW density and the output voltage. The NW density is modified by changing the width of the unit cell. A peak voltage of 0.26 V is reached for the density of around 25 µm^−2^, as a result of the balance between two main effects relating to the electromechanical coupling.

The first effect stems from the strain variation of the ZnO/matrix bottom layer ($S_{bottom}$), which substantially decreases as the NW density increases (cf. Figure 8b). Actually, increasing the density gives rise to an enhanced ZnO fraction content of the bottom layer composite, the effective Young’s modulus of which is significantly boosted because of large differences in the mechanical properties between ZnO and PMMA ($Y_{ZnO}=127\;\mathrm{GPa}$ as opposed to $Y_{PMMA}=3\;\mathrm{GPa}$). The piezoelectric voltage, ultimately related to the strain variation, drastically drops, particularly with a density higher than 50 µm^−2^. It is noteworthy that the addition of NWs in particular, or fillers in general within polymers, can lead to a significant change in their mechanical properties, which depend on the filler percentage. For instance, Chieruzzi et al. pointed out that making glass ionomer cements (GIC) with a good content of nanohydroxyapatite fillers allowed the researchers to get the best compromise between the elastic modulus, the compressive strength, and the curing time [45]. Actually, an excessive reduction of the compressive strength can induce a higher risk of failure of the restoration under normal masticatory forces. On the other hand, a reduction of Young’s modulus (such as an increase of plastic deformation) produces a positive effect on the elasticity of the material, especially when being used in a partially removed caries-based ART (atraumatic restorative treatment) technique. A more elastic material in the early stages of the procedure represents a clinical advantage in low collaborative patients for higher adhesion and moldability. In [46,47], authors reported that by incorporating the plasticizer into relaxor ferroelectric polymers, it is possible to substantially enhance their mechanical deformation, leading to higher performance in the electromechanical coupling of the blends. Those materials, however, should be polarized under high voltage to produce piezoelectricity. It is contrary to our developed materials where no polarization is needed. This fact is perhaps originated from the particular O- or Zn-polarity of the resultant nanowires, which naturally create a spontaneous polarization field [48].

The second effect is dedicated to the evolution of the effective permittivity of the bottom composite, as displayed in Figure 8c. More specifically, due to the discrepancy in the dielectric properties between ZnO ($\varepsilon_{ZnO}=12.64$) and PMMA ($\varepsilon_{matrix}=3$), a rising trend of the equivalent permittivity of composite with the increasing NW density was observed. As a consequence, the resulting output voltage somewhat improves due to the increasing proportion of piezoelectric ZnO, i.e., based on the well-known 3D electrostatic effects [49].

To summarize, the optimization of the piezoelectric response should consider those opposed tendency effects. As suggested, the ZnO NW density has a higher impact on the mechanical properties than on the electric ones. In other words, the decreasing trend of the strain is dramatically more aggressive compared to the increasing trend of the relative permittivity. Therefore, a moderate density of around 25–40 µm^−2^ seems to be the most optimized configuration.

#### 3.1.5. Influence of NW Dimensions

Besides the NW density, we demonstrate that the piezoelectric behavior also depends on the size of NW, which is assumed to be a perfectly cylindrical shape. In practice, we fabricated NWs with a dimension of around 42 nm radius and 1031 nm length. To better assess the effect of each parameter on the piezoelectric potential, two simulation trials are carried out, in which one parameter is varied while the other is fixed. In both tests, the unit cell is submitted to a static compressive stress of 1 MPa along the vertical *z*-axis.

**Figure 9:**
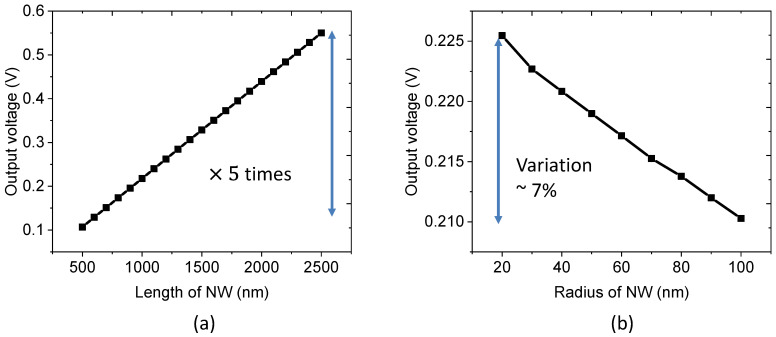
Effect of the (**a**) NW length, and (**b**) radius, on the generated output voltage subjected to a constant compressive stress of 1 MPa.

As seen in Figure 9a, for a constant radius of 42 nm and a fixed volume fraction of ZnO, the generated output voltage is proportional to the NW length, with a rate of 0.22 V/µm that corresponded to the induced electric field. As the electric field was found to be constant, an increase in the NW length leads to enhancement of the output voltage. Figure 9b shows the evolution of the output voltage as a function of the NW radius, with a fixed length of 1031 nm. In order to keep the NW density unchanged, a ratio α between the NW diameter and the unit cell width was fixed during the simulation. Interestingly, the output voltage slightly decreases as the NW radius is increased, creating a somehow non-homogeneous strain distribution between the ZnO NW and PMMA matrix. It is strongly suspected that this effect could lead to a small decrease in the strain response, and so does the output voltage. Nonetheless, with a very small variation of the output voltage (~7%) under a large radius range from 20 to 100 nm, we can conclude that the NW diameter has few impacts on the piezoelectric performance of the whole composite. This is in contrast to the NW length parameter, which can result in higher output voltage when the length is increased. This value, to some extent, should be reasonably limited so as not to weaken the NW, which can reach fracture strength under a significant dynamic action of stress.

### 3.2. Model of ZnO NW Composite

**Figure 10:**
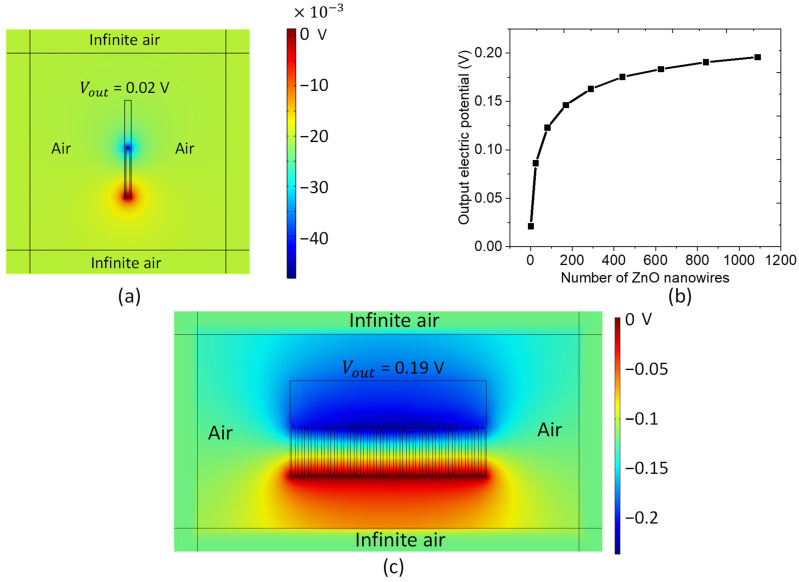
FEM simulation from: (**a**) one nanowire matrix, to (**c**) 30 cells × 30 cells nanowire matrix, subjected to an axial compressive stress of 1 MPa. (**b**) Variation of the electric potential on the top electrode as a function of the number of NWs.

In order to simulate the realistic model, the NW unit cell was embedded in an infinite air environment to provide the zero-electric-field boundary condition at a distance far away from the device (Figure 10a). Due to the strong edge effect and electrostatic effects, the output voltage of one unit cell leads to a much lower value (around 0.03 V) as opposed to the one generated from one unit cell (0.22 V), without taking into account the air effect (Figure 4b). In Figure 10b, the output voltage firstly increases with the number of ZnO NW unit cells, then gently saturates at a maximum value when the cell number exceeds 600 NWs. For a large size of 30 × 30 cells (Figure 10c), the electric potential reaches up to 0.19 V, which is close to the reference value generated in a single cell. As a result, an array with at least 25 × 25 unit cells surrounded in air is regarded as a suitable model to predict the theoretical performance in comparison with the experimental results.

**Figure 11:**
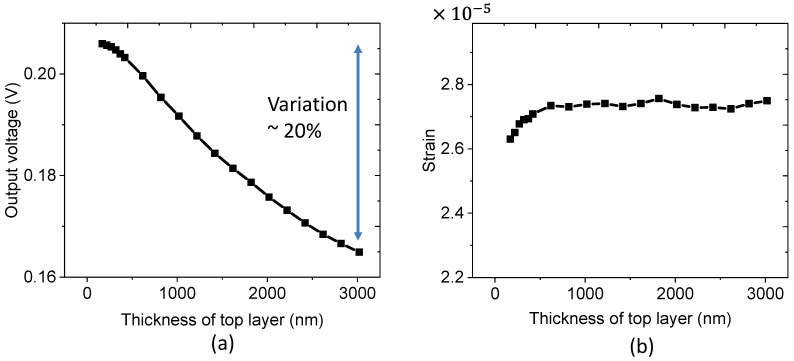
FEM simulation results of the effect of top layer thickness on the (**a**) generated output voltage, and (**b**) the strain of ZnO.

During the fabrication of the vertically aligned ZnO NW array/polymer composite, the thickness of the top layer was a key parameter and was required to be determined in advance. As suggested in the FEM simulation illustrated by Figure 11a, the resulting voltage generated from the array matrix gradually decreases as the thickness of the PMMA top layer is increased. On one hand, increasing the PMMA thickness leads to less ZnO concentration in the samples, provoking a decline in the piezoelectric response. More specifically, the decrease of the potential merely arises from the fact that electric potential or field at a position generated from the charge, regarded as the top of the NW here, decreases with the distance between them in classical electrostatics [50,51]. On the other hand, the mechanical strain of the ZnO NW composite seems to be stable as a function of the PMMA thickness (cf. Figure 11b); only very little fluctuation has been observed for the whole thickness range from 500 nm to 3000 nm. It is therefore permitted to conclude that Young’s modulus of the composite is almost unchanged, regardless of the top layer thickness. Similar to what was observed in Figure 5d, a slightly increased slope occurs at the small thickness of the top layer (i.e., 250–500 nm, Figure 11b), which is due to the non-homogenous structure of the bottom composite. Consequently, the decreasing potential is not related to strain variation, but is rather caused by the diminution of the electric field generated within the ZnO NW.

Finally, the utilization of the COMSOL simulation in this work provides basic optimization guidelines for the development of a vertically aligned ZnO NW/polymer composite. This step allowed us to better understand the effects of the material properties (Young’s modulus, relative permittivity), as well as the geometric design of the structure (NW shape and density, top layer thickness), on the piezoelectric sensor performance. However, in comparison with the actual experimental performance, the simulation still ignores several considerations, like the semiconducting properties of ZnO, the dielectric loss in the dielectric material, and so on. The following section will clarify this issue.

## 4. Results and discussions

### 4.1. Morphological Properties of as-Grown ZnO Nanowires

**Figure 12:**
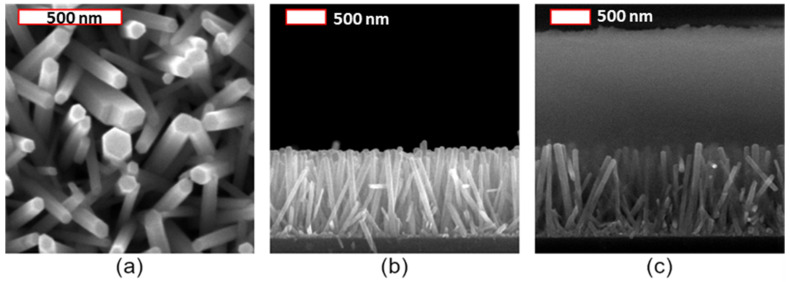
FESEM images of (**a**) top view and (**b**) cross-section view of ZnO NWs grown by CBD. Image (**c**) depicts the ZnO NW arrays, with a PMMA matrix exhibiting a thickness of ~1.5 µm in the top.

The morphology of ZnO NWs was assessed by field-emission scanning electron microscopy (FESEM) imaging, using a FEI Quanta 250 FESEM instrument. The coating of (001) Si substrate with a 40 nm-thickness ZnO SL was achieved by a dip-coating process on a sol-gel solution. The polycrystalline ZnO SL was strongly oriented along the polar c-axis, and ZnO NWs by CBD are known to grow homoepitaxially on top of it [52]. The CBD growth was performed with an equimolar precursor solution of Zn(NO_3_)_2_ and HMTA at 85 °C, and with a pH value of around 5.5 [53,54]. The hexagonal shape, typical of the wurtzite structure, is presented in Figure 12a by FESEM imaging, showing that they are oriented along the polar *c*-axis. According to Figure 12a,b, ZnO NWs exhibit a mean radius and length of 42 ± 8 nm and 1031 ± 16 nm, respectively. Additionally, Figure 12c depicts the ZnO NW arrays, encapsulated in a PMMA matrix deposited by spin-coating, and exhibiting a thickness of ~1.5 µm on the top.

### 4.2. Electrical Properties

This study details the electric characterization of our designed devices, which is represented by the current density–electric field (J-E) curve. The first column of Table 1 shows different materials tested on the experimental setup as described in Section 2.2.2. To better understand the electrical behavior of the complex structure (consisting of 4 layers like a Si substrate, ZnO seed layer (SL), ZnO NWs embedded in PMMA matrix, and top PMMA layer), simplified structures were investigated (e.g., with 1, 2, or 3 layers). All samples were subjected to an alternating electric field of 1 kHz frequency, and under a static stress of 1 MPa.

**Figure 13:**
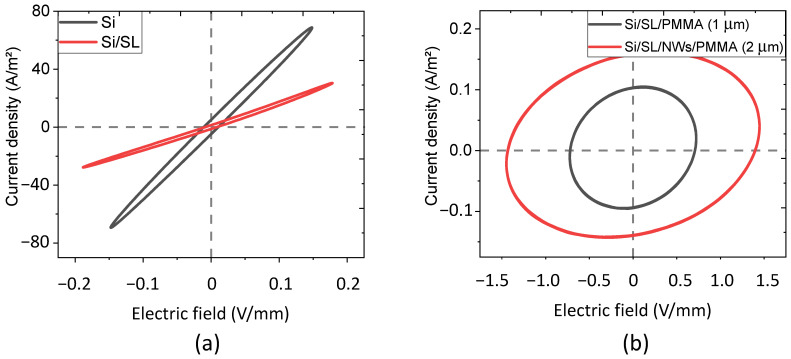
Representative current density versus the electric field of (**a**) resistive-like samples including Si and Si/SL; (**b**) capacitive-like samples including Si/SL/PMMA (1 µm) and Si/SL/NWs/PMMA (2 µm).

As expected from Figure 13a, the characteristic J-E curves of Si and Si/SL samples were perfectly linear and symmetric, which was indicated by a passive resistive behavior. The values of their resistivity, deduced from the J-E slopes, are displayed in Table 1. Logically, the bilayer Si/SL sample exhibits a superior resistivity than the pure Si sample, due to the addition of one more resistive layer of 40 nm thickness. Figure 13b shows a circular-shaped J-E curve of the 3-layer and 4-layer samples containing the PMMA, reflecting that the capacitive property is dominant with respect to the resistive one. Interestingly, increasing the thickness of the PMMA layer from 1.5 µm to 2 µm in a vertically aligned Si/SL/NWs/PMMA composite leads to moderate improvement in the permittivity (Table 1). From the surface capacitance value, it is possible to determine the equivalent dielectric permittivity of the capacitive materials. Since there is no difference in the electrical properties between the Si/PMMA (1 µm) and the Si/SL/PMMA (1 µm) samples, the only result of the Si/SL/PMMA (1 µm) is displayed in Figure 13b, i.e., overlapped. It should be noticed that, in order to improve the accuracy, all parameters in Table 1 are the average values calculated on at least five assays of the alternative input.

**Figure 14:**
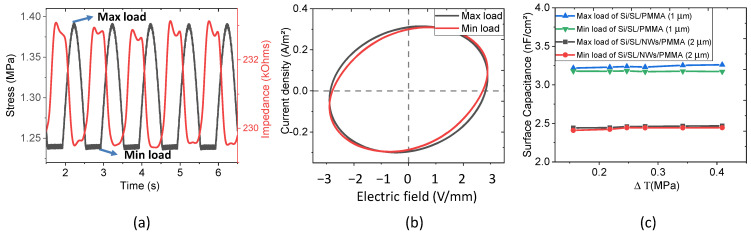
Electric characterization under a dynamic stress of 1 Hz and 0.15 MPa was carried out by applying a 1 kHz sinus electric field. For the Si/SL/NWs/PMMA (2 µm) sample, (**a**) the variation of stress and impedance with time, and (**b**) at max and min load configurations, the current always circularly changes with the electric field. (**c**) The relative permittivity of the Si/SL/PMMA (1 µm) and Si/SL/NWs/PMMA (2 µm) sample is almost constant, regardless of the dynamic stress level.

With the aim of evaluating the electrical properties under dynamic mechanical excitation, samples were subjected to a 1 Hz compressive stress, with magnitude $\Delta T=max_{load}-min_{load}$ For the sake of simplicity, only the Si/SL/NWs/PMMA (2 µm) is presented in Figure 14a,b. Figure 14a describes an inverse variation between the stress and the electrical impedance of the sample, which was caused by a slight change in the sample’s thickness during mechanical solicitation. Concretely, a peak of stress (i.e., “max load”) corresponds to the minimum impedance as the sample thickness somewhat decreases due to the compression. On the other hand, “min load” leads to the maximum impedance as the sample is “less compressed”. Interestingly, as displayed in Figure 14b, the current density still has a circular response with the electric field regardless of variation in the mechanical excitation. When slightly increasing the dynamic stress (∆T from 0.1 to 0.4 MPa), the surface capacitance of Si/SL/NWs/PMMA composites and the Si/SL/PMMA samples was almost constant (Figure 14c). Accordingly, the capacitive behavior of the Si/SL/NWs/PMMA composite is confirmed to be stable under a dynamic uniaxial compression of less than 0.4 MPa, and so is the dielectric property. Two main reasons explain why we did not perform experiments beyond 0.4 MPa:

- The Si substrate was rigid, fragile, and could be easily broken under significant mechanical solicitation. The flexible substrate-based polymer matrix, in which grown ZnO NWs are currently under investigation by our team in order to adapt them for medical use as a flexible sensor catheter, is required.
- Knowing the maximum blood pressure (systolic) in large human arteries is 100–150 mmHg (~13–20 kPa), we can conclude that 0.4 MPa is largely enough to test an application for FFR.

### 4.3. Dielectric Properties

**Figure 15:**
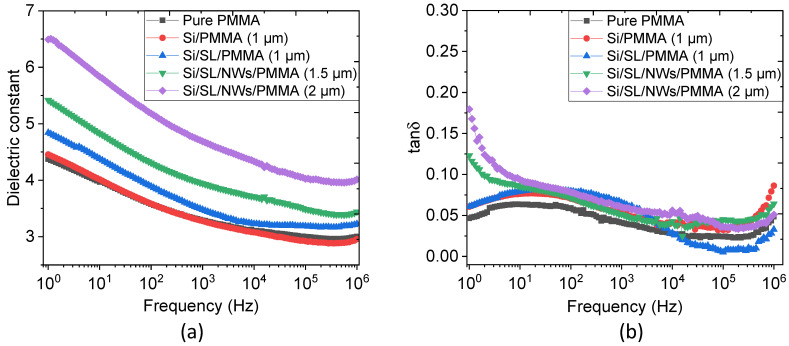
Variation of the (**a**) dielectric constant, and (**b**) loss tangent as a function of the frequency of different samples.

In order to further elucidate the dielectric properties of capacitor-like samples, the variation of the dielectric constant (ε) and loss tangent (tanδ) versus a frequency ranging from 1 Hz–1 MHz were plotted in Figure 15. For easier comparison, a pure PMMA sample, chosen as a reference sample, was fabricated in our lab based on the thin film casting method [33,34]. It was expected that the two “passive” samples (red and blue lines) would exhibit similar dielectric behavior with respect to the pure one. Whatever the frequency range, both Si/SL/NWs/PMMA composites led to an improvement in the relative permittivity, which may be due to the fact that the PMMA polymer might penetrate and sediment into the interstitial space between ZnO NWs. As observed in Figure 15b, the superior tanδ loss of Si/SL/NWs/PMMA composites occurs at a low frequency range, which probably originates from the introduction of interfacial polarization between the NWs and the polymer matrix.

### 4.4. Piezoelectric Properties

**Figure 16:**
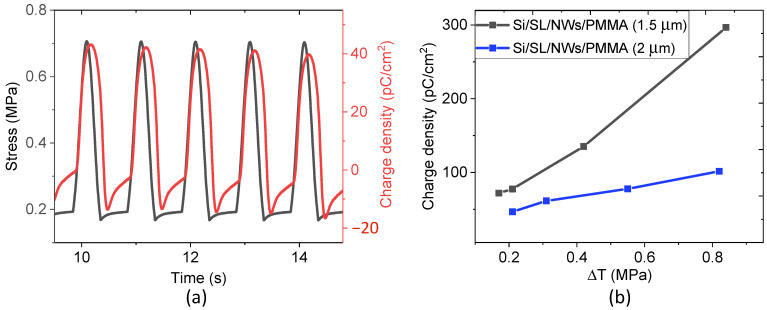
Piezoelectric characterization, performed under a dynamic stress of 1 Hz. (**a**) Time evolution of stress and charge density of the Si/SL/NWs/PMMA (2 µm) composite. (**b**) Piezoelectric charge density versus the dynamic stress of samples with different thicknesses of the top PMMA layer.

As described in Figure 3, by applying dynamic mechanical stress on the sample in a short circuit, the electric charge can be collected; the piezoelectric coefficient d_33_ was then evaluated. Experimental tests were performed on the two “active” samples, consisting of ZnO NW arrays embedded into a PMMA matrix designed with different thicknesses of the dielectric top layer (i.e., 1.5 µm and 2 µm). The remaining “passive” samples, from which no charge signals were observed under mechanical excitation, are not worth presenting here. Figure 16a describes the piezoelectric behavior of the Si/SL/NWs/PMMA (2 µm), where the periodic variation of the generated charge density is in accordance with the applied stress. This result confirms the piezoelectric effect of the ZnO NW array/PMMA composite described in Equation (3), where the electric charge density (*D*) was perfectly in phase and exposed a similar trend with respect to the input of mechanical stress (*T*). It is expected in Figure 16b that the variation of *D* linearly increases with the increasing Δ*T*, allowing an estimation of the piezoelectric coefficient (d_33_), deduced from the slope of the D-versus-ΔT characteristics. It has been pointed out that the Si/SL/NWs/PMMA (1.5 µm) gives rise to an approximately threefold improvement in the piezoelectric response (d_33_~3.53 pC/N), compared to the 2 µm counterpart (d_33_~1.21 pC/N).

**Figure 17:**
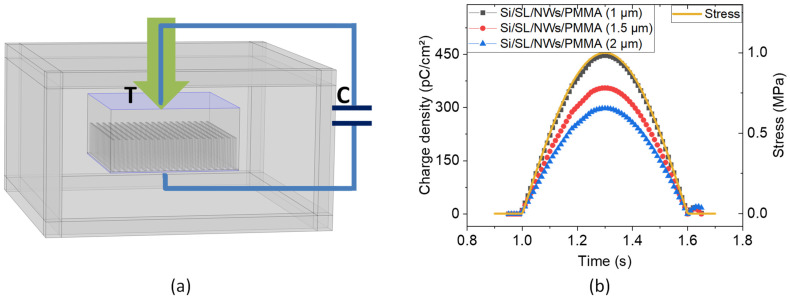
FEM simulation performed using the “Piezoelectricity module” coupled with “Electrical circuit module”; (**a**) schematics of the ZnO NW composite, in parallel with an external capacitor of 100 nF subjected to a compressive stress of 1 MPa magnitude; (**b**) theoretical piezoelectric response of different NWs/PMMA composite with respect to the PMMA thickness.

It should be noted that, in a linear regime (i.e., subjected to moderate mechanical stress), the effective piezoelectric coefficient of the composite does not depend on the input compression, but rather on the intrinsic properties, such as the designed structure and material properties. Therefore, to estimate the d_33_ of a single ZnO nanowire, FEM under an arbitrary compression was performed to describe the effective piezoelectric response of the composite. In COMSOL Multiphysics, the piezoelectricity module and electrical circuit module were coupled with each other by applying proper terminal conditions. Specifically, a capacitor was connected in parallel to the composite, so as to collect and accumulate the piezoelectric charges in response to compressive stress. This method allowed us to easily estimate the effective piezoelectric constant (d_33_) along the vertical, as illustrated in Figure 17a. With the abovementioned approach described in the modeling (Section 3.2, Figure 10), 25 × 25 periodic unit cells embedded in the finite air were chosen to avoid the edge effects.

The study was carried out in the time domain in which the composite was excited on the top by a positive sinus compressive stress of 1 MPa amplitude. In order to achieve the best fitting between the experimental and simulation results, the piezoelectric coefficient of each ZnO NW was set at approximately d_33_ = 16.5 pC/N. It is highlighted in Figure 17b that when the stress increases, the composite generates increasing electric charges on the electrodes, which are then stored in the external capacitor. On the other hand, the accumulated charges start to decrease when the composite is mechanically released. Table 2 compares the experimental and simulated piezoelectric coefficient of ZnO NW composites with different thicknesses of PMMA. Under identical compression mode, the composite with a thinner top PMMA layer provides higher instantaneous surface charges, giving rise to a significantly improved piezoelectric coefficient (d_33_).

The result of Table 2 clearly shows a discrepancy between experiment and simulation in the piezoelectric response. Interestingly, this discrepancy appears for the 2 µm-thick PMMA but not for its 1.5 µm counterpart. Accordingly, the difference between the model and the practice might be affected by the thickness of the top dielectric layer itself. A similar result can be found in the literature [23,50]. Another reason could be due to the fact that, in the simulation model, several factors (such as the screening effect in the ZnO semiconductor, caused by free charge carriers, dielectric loss in the material, conduction loss in the circuit, etc.) are not considered, for the sake of simplicity. Therefore, it could be concluded that the d_33_ coefficient of the ZnO NW used in the simulation (16.5 pC/N) should be lower than the real value. It is worth noting that the sample with the PMMA thinnest top layer (1 µm) exhibits unexpected behavior, and thus makes the charge measurement unsuccessful. Actually, such a thin layer might create a short-circuiting effect or some kind of defect on the surface, provoking current leakage in the material and making it behave as a resistor instead of a capacitor. Therefore, in reality, a thickness of 1.5 µm for the PMMA top layer seems to be the best compromise to achieve a satisfactory piezoelectric response.

## 5. Conclusions

In this work, we firstly performed FEM simulations to better understand the influence of various relevant parameters on the piezoelectric response of Si/SL/NWs/PMMA composite. The main objective of this task involved achieving an optimal design structure, which relied on the material properties and geometries so as to meet a high sensing performance that could be adapted to biological media in future applications [55,56,57]. The simulation results revealed that the mechanical Young’s modulus, as well as the dielectric permittivity of the PMMA bottom matrix, had a high impact on the output voltage, contrary to those of the top layer, where no worthy effect has been observed. Moreover, NW characteristics, comprising density, length, and radius, were also considered to get the best trade-off between the mechanical behavior and the piezoelectric sensor response.

Another objective of this work reported on experimental tests, with the aim of identifying the characteristics of each layer as well as of the whole complex structure. Electrical characterization investigated a set of designed configurations, allowing us to confirm that the silicon and ZnO seed layer performed like a resistor, while the other samples stacked with a PMMA layer behaved like a capacitor. In addition, we demonstrated that the dielectric properties of these capacitors were perfectly stable under a dynamic stress varying from 0.1 to 0.4 MPa. A small change in their electric impedance was originated from the thickness variation when the sample was subjected to a compressive force. Experimental measurements highlighted that a ZnO NWs composite with a lower thickness of PMMA top layer gave rise to increased piezoelectric sensitivity. The effective charge coefficient (d_33_) of the fabricated composite reached 3.53 pC/N when being stacked with a 1.5 µm-thick PMMA, as opposed to 1.21 pC/N in the case of the 2 µm-thick PMMA counterpart. This effect to some extent correlated with the model predicted by COMSOL FEM. Accordingly, significant improvements in piezoelectric behavior were achieved through the optimization of the top-layer thickness (i.e., 1.5 µm). Furthermore, thanks to novel material design based on the vertically aligned ZnO NW arrays, it allowed us to notably boost the piezoelectric sensitivity, being seven times superior to the ZnO particle composite developed in our previous work [34,41].

These results are very promising, confirming that a ZnO NW composite, with the further benefit of no polarization being needed, can reach a comparable piezoelectric performance to conventional alternatives like BaTiO_3_. In the near future, vertically ZnO NW arrays integrated into a flexible substrate will be one of our main concerns. The goal of this research work aims to validate the feasibility of self-powered piezoelectric sensors for implantable biomedical detection, particularly for the FFR technique.

## Figures and Tables

**Table 1:** Electrical measurement of different samples.

Sample	No. of Layers	Resistivity (Ω∙m)	Surface Capacitance (nF∙cm^−2^)	Relative Permittivity	Property
Si	1	2.144 ± 0.004	-	-	resistive
Si/SL	2	6.321 ± 0.009	-	-	resistive
Si/PMMA (1 µm)	2	-	3.212 ± 0.005	3.809 ± 0.006	capacitive
Si/SL/PMMA (1 µm)	3	-	3.195 ± 0.003	3.760 ± 0.004	capacitive
Si/SL/NWs/PMMA (1.5 µm)	4	-	3.089 ± 0.009	5.108 ± 0.015	capacitive
Si/SL/NWs/PMMA (2 µm)	4	-	2.439 ± 0.004	5.509 ± 0.009	capacitive

**Table 2:** Piezoelectric coefficient d_33_ from experimental and COMSOL simulation.

	Measured Effective d_33_ (pC/N)	Simulation Effective d_33_ (pC/N)
Si/SL/NWs/PMMA (2 µm)	1.21 ± 0.16	2.98 ± 0.03
Si/SL/NWs/PMMA (1.5 µm)	3.53 ± 0.33	3.55 ± 0.01
Si/SL/NWs/PMMA (1 µm)		4.46 ± 0.05
